# Online and blended entrepreneurship education: a systematic review of applied educational technologies

**DOI:** 10.1007/s41959-021-00047-7

**Published:** 2021-04-16

**Authors:** Li Chen, Dirk Ifenthaler, Jane Yin-Kim Yau

**Affiliations:** 1 University of Mannheim, L4, 1, 68161 Mannheim, Germany; 2 Curtin University, Perth, Australia

**Keywords:** Entrepreneurship education, Blended and online learning, Social media, Serious games, Massive Open Online Courses

## Abstract

The supply and demand of entrepreneurship education at university level commenced in 1938. With the proven entrepreneurial effectiveness in economic development and the efforts of scholars, policymakers and other stakeholders, competencies in entrepreneurship are becoming a set of essential learning objectives. In the digital era, entrepreneurship education can be made available in an online and blended format. Thereby, this study presents a systematic analysis of research focusing on blended and online entrepreneurial learning and teaching. Based on five keywords, collating an initial set of 121 articles, this systematic review details the research outcomes of the resulting set of 38 published research articles/contributions, where each described a specific online and blended learning environment. We obtained and analyzed the following information from each of these articles: definition of entrepreneurship education, context of study, methodology, applied technology, focused group, sample, outcome of entrepreneurship education and research rigor. Our findings showed that the current research status and achievements scholars have contributed in educational technologies utilized by online and blended entrepreneurship education can be summarized into three categories: social media, serious games and Massive Open Online Courses. In order to compare these technologies, we selected five examples from three educational technologies and utilized a marking sheet for evaluation and assessment. In general, it was found that Wiki was used to discuss entrepreneurial concepts and that Facebook was the most common social software in entrepreneurship education. In terms of serious games, *FLYGBY* and *SimVenture* facilitated the gamification and enjoyment of entrepreneurship activities the most. Finally, as Massive Open Online Courses platform, Coursera offers plenty of/online entrepreneurship education courses. In a nutshell, in online and blended entrepreneurship education, social media was utilized to facilitate cooperation amongst participants; serious games were used to enhance students’ enjoyment and engagement; and Massive Open Online Courses provided a platform as well as high-quality learning resources, anywhere anytime. Hence, each technology has advantages and challenges when we apply it to entrepreneurship education. We conclude that instructors and learners need to successfully compare and choose the most appropriate combination of technologies to achieve entrepreneurial course aims.

## Introduction

The COVID-19 pandemic started in 2020 that has increased the speed in the facilitation of online and blended education, especially in higher education institutions, as the education of university students in developed countries has been moved online and will continue to be online until the pandemic is over. This created an obstacle for the provision of entrepreneurship education (EE) as it is a discipline, which requires students to acquire “learning by doing”—practical competencies and experiences in an authentic setting (Liguori & Winkler, 2020; Kassean et al., 2015; Kuratko, 2005). However, due to the COVID-19, educators need to transfer offline educational activities into the online domain. To make this transition as seamless as possible and ensuring that the teaching and learning objectives are met, the emphasis was placed on the utilization of online and blended applications/technologies. Although there have been reviews on various technologies that have been applied in business education including a review of social media (Tess, 2013), a review of serious games (SGs) (Faria et al., 2009) in this context, as well as a general review of online and blended education (Arbaugh et al., 2009), there have been limited reviews conducted on the utilization and effectiveness of educational technologies in EE (Fayolle, 2013; Rashid, 2019). Various reviews of specific technology can be found; for example, Fox et al. (2018) built a criteria framework to review and evaluate serious entrepreneurial games; educational technologies applied to online and blended EE include technology-mediated to an intelligent method, e.g., *computer aided instruction* (CAI) (Petridou & Sarri, 2011), *information technology* (Nisheva et al., 2009) *virtual and augmented reality* (Sousa, 2019; Tarabasz et al., 2018), *and big data* (Obschonka & Audretsch, 2020). Our review of the articles which utilized technologies in EE encompasses the following: *blog* (Wagid & Oliver, 2017; Udosen & Upula, 2019), *Wikis* (Menkhoff & Bengtsson, 2012), *Facebook* (Chang & Lee, 2013; Ali et al., 2017), *digital and non-digital serious games*, *course management system* (Frederick, 2007; Wu et al., 2019) and *MOOCs* (Cirulli et al., 2016; Resei et al., 2018; Vorbach et al., 2019).

Our research is motivated by first the lack of a systematic review on how educational technologies have been effectively applied in EE and second the lack of information on the new technologies that have already been introduced to existing EE courses. One of the aims of this systematic review is to provide guidelines for informing decision-makers and educators about the advantages and challenges of the utilization of these technologies for EE and for supporting them in selecting appropriate tools for their courses. The reminder of the paper is divided into the following—a literature review is presented in “Literature review” section, our research methodology and questions are presented in “Research methodology” section, the results of our systematic review are presented in “Results of the systematic review” section, and thereafter a discussion and implications of this research in “Discussion” section followed by the conclusion in “Conclusion” section.

## Literature review

A number of authors have presented research studies on entrepreneurship intention (such as Bae et al., 2014; Ngoc Khuong and Huu An 2016) and their implication (such as Henry et al. 2005; Oosterbeek et al. 2010); however, research studies on educational technologies in EE have been limited despite the practical development and presentation of online and blended EE courses using the internet and educational tools since the last twenty years. Currently, technologies such as Web 2.0, cloud computing and artificial intelligence have been utilized for supporting blended and online entrepreneurship teaching and learning. Student management and response systems (e.g., Moodle and Business Operation Support System) have been used to support learning and teaching as well as collect and analyze learning behavior data. Devices such as laptop or tablet computers, mobile devices and smartphones are used as a medium for teaching and learning entrepreneurial knowledge, skills and competencies. Many studies have been presented, which adopted different technologies in online and blended EE such as Web 2.0 (Jones & Iredale, 2009), cloud computing (Holinska et al., 2019; Ratten, 2013), digital technology (Rippa, 2018), Massive Open Online Courses (MOOCs) (Al-atabi & Deboer, 2014; Chang, 2017; Cirulli et al., 2016; Resei et al., 2018; Vorbach et al., 2019), social media (Ali et al., 2017; Chang & Lee, 2013; Waghid, 2017) and Serious Games (SGs) (Protopsaltis et al., 2014; Romero & Usart, 2013). The last three mentioned technologies have been broadly (in relative terms) adopted in online and blended EE. Educators typically adopt more than one technology for the implementation of their EE courses such as Facebook as the type of social media utilized on Moodle 2.0 as the learning management system. An EE course combining SGs within a learning platform has been presented by Protopsaltis et al. (2013) and one combining SGs with social media has been presented by Wu and Song (2019). In order to compare different combinations of technologies deployed in EE courses, a definition of EE needs to be specified, which is detailed in the next section.

### Entrepreneurship education

EE is rarely defined or conceptualized (Fayolle, 2013). Based on the definitions from Sexton and Bowman (1984), Gibb (2002), Rasmussen and Sørheim (2006) and Liñán (2004), EE consists of learning activities, which allow learners to acquire entrepreneurial knowledge, skills and attitudes necessary for creating and operating a business. The Global Entrepreneurship Index Report 2018 (relating to the entrepreneurship ecosystem) highlighted that the Global Entrepreneurship Index scores have increased by 3% worldwide (Acs et al., 2018). It showed that North American and European account for 15 occupations in the top 20. The performance of EE presented a similar distribution. Commonly, EE originated from the USA and become a mainstream discipline in business schools as well as other schools in higher education institutes (HEIs), partly because innovation is the most consequential characteristic in American culture, education and society, which meets the requirements of EE (Brooks et al., 2019). Due to various existing welfare systems and cultures, entrepreneurship and EE in Europe lag behind those of the USA (Potter et al., 2008; Karimi & Chizari, 2010). Therefore, European policy decision-makers executed many initiatives to chase the trend. The implementation of the Bologna Process (Keeling, 2006) facilitated European universities and colleges to be more innovative and entrepreneurial (Potter et al., 2008). The European Commission built an entrepreneurship competence framework containing three competence areas and 15 specific competencies to guide entrepreneurial academics and actions (Bacigalupo et al., 2016). Australia ranked first in the Asia–pacific area in the 2018 report and offered 584 entrepreneurship subjects in 2015 (Maritz et al., 2015). 70% of Malaysian HEIs have built entrepreneurship incubators, and they offered entrepreneurship activities in almost every university (Cheng et al., 2009; Rahim et al., 2015). Chinese Ministry of Education takes the entrepreneurial course as a general and compulsory course in higher education institutes.

EE plays an important role at different stages of education; however, current EE courses as well as researches are typically available and popular in HEIs (or business and management schools) as under-, post-graduate degrees or MBAs. Many researchers attempt to answer “Why,” “How,” “What,” “Who,” “When,” (von Graevenitz et al., 2010; Lackéus & Middleton, 2015) and “Where” questions related EE (Karimi & Chizari, 2010; Zhou & Xu, 2012). A number of empirical studies of EE have been conducted including Fox et al. (2018) and Wu et al. (2018) as well as meta-analyses conducted by Martin et al. (2013), Schlaegel and Koenig (2014), and Bae et al. (2014). These studies were conducted from a variety of disciplines such as business, education, engineering and computer science.

### Educational technologies deployed in EE

“Educational technology is the study and ethical practice of facilitating learning and improving performance by creating, using and managing appropriate technological processes and resources” (Januszewski & Molenda, 2013). As the routine of pedagogy of EE is from teacher-led to student-centered (Robinson et al., 2016) and constructivism (Löbler, 2006), the tendency of educational technologies changes from teaching design to learning environments (Januszewski & Molenda, 2013). Namely, the key objective of entrepreneurial educational technologies is to facilitate active, intentional, constructive and collaborative learning.

Many different but similar concepts of learning environments were utilized; for example, Moore et al. (2010) argued that the analysis of various ingredients of learning environment was essential. According to the percentage of technologies used in education, 30–79% consisted of blended courses and 80+% consisted of online ones (Allen & Seaman, 2008). Siemens and Tittenberger (2009) noted that EE utilized additionally augmented technology extending the classroom, blended and online learning approaches. Watson (2008) argued that blended learning was a connection between F2F (face-to-face) and fully online learning. Online learning is considered as the utilization of the Internet and a computer to deliver courses. Therefore, the definitions of learning environments have not been unanimously endorsed. When a definition of online and blended learning was required for the application of EE courses, researchers tended to adopt their self-definition in their research (namely descriptive definition). Bonk and Graham (2004) argued that blended (hybrid) learning was a combination of F2F learning and distributed learning, which is centered on computer or mobile technologies. Course designers adopt both online and offline activities in a real or virtual classroom through using synchronous and asynchronous educational technologies (Frederick, 2007).

Compared with F2F EE courses, educational technologies are indispensable when students participate in entrepreneurship activities in online and blended entrepreneurial learning environments. Besides, educational technologies bring with the trend that online and blended entrepreneurial courses are becoming one of the main choices for students, educators and enterprises. Furthermore, a large number of applications applied to EE are produced and updated (e.g., Second Life, Facebook and online forums), because developers customize certain education technology to meet the needs of stakeholders, while studies of entrepreneurial educational technologies are scattered and systematic reviews on this topic are lacking. Due to time and resource constraints, developers of educational technology focus on one or two technologies, develop and experiment with one system or application (Buzady & Almeida, 2019). Researchers do not tend to compare the effectiveness of two or three technologies. Besides, educators and learners need to understand the advantages and challenges of technologies, which is a basis for choosing a suitable one for learning and teaching. Therefore, conducting a systematic review and comparing the educational technologies utilized in EE are essential for combining theory and practice to construct a successful EE course.

### The difference of entrepreneurship teaching and learning

Entrepreneurship pedagogy, the effectiveness for sociality and the economy are the main objectiveness for EE (Fayolle, 2008). Concerning entrepreneurship pedagogy, namely entrepreneurship teaching and learning, the two concepts are defined for it to be understood clearly. Here, we analyzed both from the objective, research, method and evaluation aspects. EE is not directly aimed at increasing the number of start-ups and entrepreneurs, but enhancing the life-long skills that a graduate needs for undertaking business endeavors or finding an occupation in the future (Jones, 2010). Clearly, entrepreneurship teaching aims to deliver entrepreneurship knowledge, convey entrepreneurship skills and teach students how to start a business (Gibb, 2002). As entrepreneurship learning relates to individuals and their backgrounds, there is a gap between teachers’ teaching and learners’ learning. Teacher’s self-learning and reflection processes affect entrepreneurship teaching (Seikkula‐Leino et al., 2010), while factors from the learners’/learning side are more sophisticated, such as their age (Honjo, 2004), education (Barringer et al., 2005), family entrepreneurs (Wadhwa & Aggarwal, 2009) and personality traits (Barkham, 1994).

There are several traditional and non-traditional methods related to EE: lectures, guest speaker, action-based entrepreneurship programs (esp. workshop, study visits, counselling, setting up a business, games and practical training) (Hytti & O’Gorman, 2004; Rasmussen & Sørheim, 2006). In theory, every method is equal to be introduced into the entrepreneurship class. In practice, learner’s preferences and experiences affect learning method choice. As one assumption said: “students will encounter a similar set of circumstances in the future for which they will be better prepared” (Fiet, 2001), instructors’ teaching design applied simplified and generalized entrepreneurship process. However, the authentic entrepreneurship learning environment is vague and complicated, since learners learn from daily life as well. Entrepreneurship learning is the core aim to evaluate EE in all learning levels. The top five variances measured in entrepreneurship programs: perceptions, attitude, self-efficacy, entrepreneurial orientation and creativity that are related to learners. And studies of “training” only occupied 7% (Huang-Saad et al., 2018). That means the number of qualified and trained teachers, the number of courses and programs, and teacher activities (Vesper and Gartner, 1997; Purzer & Fila, 2016) are less mentioned. In the end, entrepreneurship teaching and learning are different when adopting educational technologies, which we discuss in the “results” and “discussion” sections. To advance the effectiveness and efficiency of EE, basing on the student-centered concept, our scholars, policy-decision makers and stakeholders need to focus on entrepreneurship learning.

## Research methodology

### Research questions

We conducted a systematic review and compared the utilized technologies from three aspects—pedagogy, usability and technological. Five concrete examples have been selected for analysis and comparison in detail. The research questions are:

1. How are SGs, social media and digital platforms (mainly MOOCs) technologies applied in online and blended EE?
2. What are the strengths and weaknesses of SGs, social media and digital platforms (mainly MOOCs) in online and blended EE?

### Methodology

**Fig. 1:**
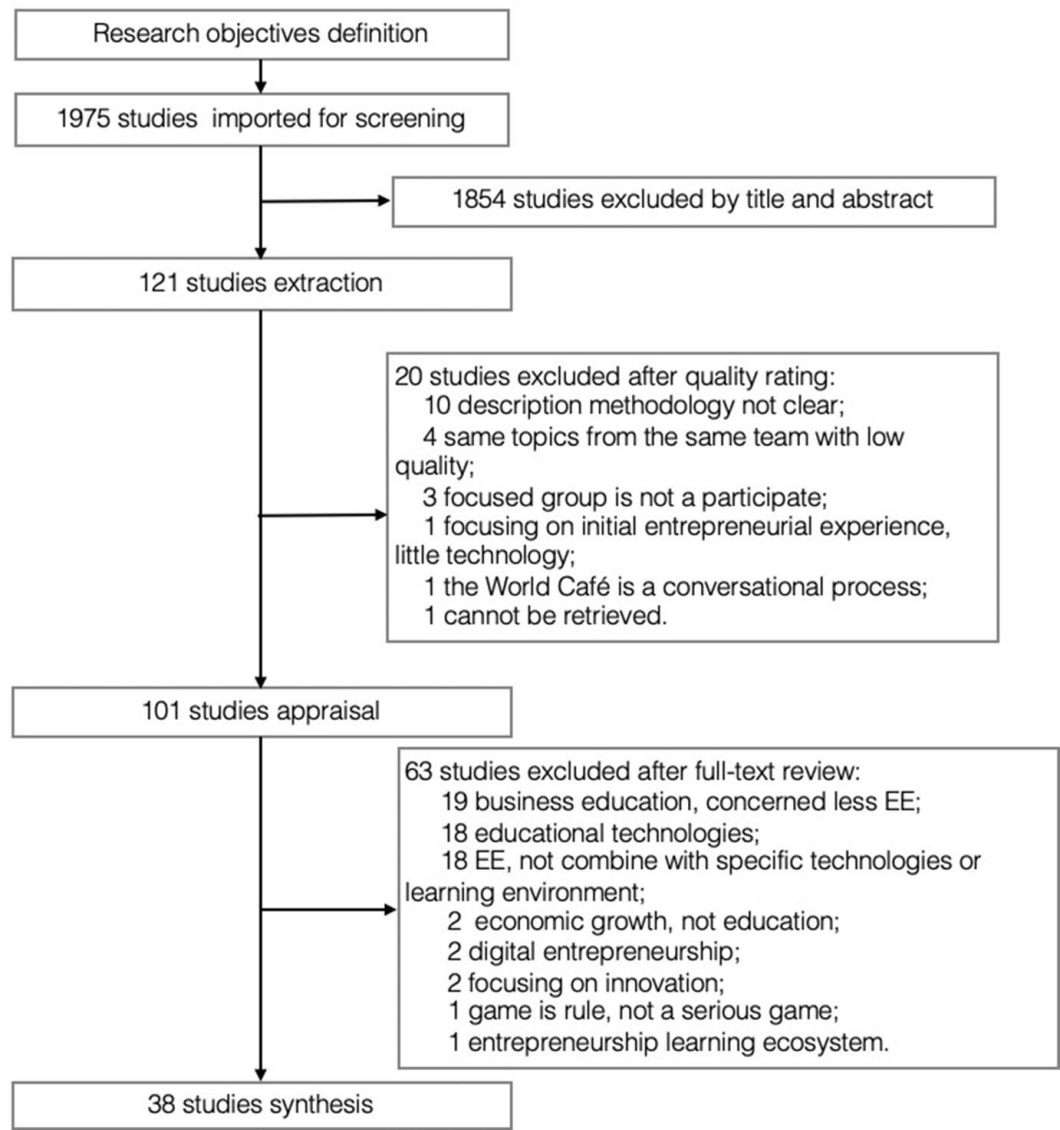
Steps of literature collection

The main research objective was to conduct a systematic review, based on Okoli’s (2015) eight steps, of the application of educational technologies in online and blended EE. The articles under review were limited to the last twenty years (2000–2020) and in the English language. The focus was on “educational technologies” with “entrepreneurial education” and not “technology entrepreneurship”, “university incubator” and “technology transfer”. The utilized keywords included “entrepreneur* education,” “education technology*,” “blended,” and “online”. We searched journals of high impact factor in EE such as Journal of Business venturing, The Piccola Impresa/Small Business Journal, Education + Training, Technovation, International Small Business Journal: Researching Entrepreneurship, Academy of Management Learning and Education, Entrepreneurship Theory and Practice, Journal of Small Business Management and International Entrepreneurship and Management Journal (Fig. 1).

We also searched Google Scholar, Web of Science and ScienceDirect as well as 7 MOOCs platforms to collect the description of EE courses. Focusing on technologies in EE such as “distance learning,” “blended learning,” “online learning,” “e-learning,” and “mobile learning” as well as relevant technologies such as “Web 2.0,” “Wiki,” “Information and communication technology (ICT),” “MOOCs,” “social media,” and “SGs”. The string search combination showed another 121 articles between 2006 and 2020. 61 of the 121 studies were excluded after full-text review due to irrelevance. We conducted a quality appraisal step based on checklists of analyzing research quality (O’Brien et al., 2014; Tong et al., 2007; Mager & Nowak, 2012) with the following quality criterion: the description and appropriateness of clear research questions, sampling selection, data collection, data analysis and synthesis. The end result was 38 high-quality studies to be analyzed in our systematic review and classified into three categories: Social media, SGs and digital platforms (mainly MOOCs).

## Results of the systematic review

The collected 38 articles were divided into specific adopted technologies: social media (9), SGs (20) and MOOCs (9) are listed in Table 1. Every study was scrutinized from the definition of entrepreneurship education, the background of the study, methodology, applied technology, focused group, sample, the outcome of entrepreneurship education and research rigor. The literature was cited 25 times (SD = 28, Min = 1, Max = 133) on average. The studies are scattered in Italy (5), USA (4), Taiwan, China (4), Malaysia (4), UK (4), Germany (2), Greece (2), Spain (2), Austria (2), Holland (1), Hungary (1), Switzerland (1), Romania (1), South Africa (1), Portugal (1), Ireland (1), Indonesia (1) and Russia (1). Research rigor was measured from the rigor of theory background, method, result, discussion and conclusion. In terms of research rigor, we rated the articles as strong (3), moderate + (7), moderate − (20) and weak (8) levels.

**Table 1:** Systematic review applied educational technology in entrepreneurship education

	Reference	Educational technologies	Definition of EE	Context	Focused group	Sample	Method	Expected outcome of EE	Research rigor
Social media	Waghid and Oliver (2017)	Film, blogs and Blackboard	Economic and social development; address social injustices	Social entrepreneurship is not adequately; partially addressed into the curriculum for preservice educators	A cohort of 3^rd^-year Bachelor of Education	48	Case study; online group interviews	Sense of social entrepreneurship theory; practical knowledge to help deal with social injustices	Weak
	Chang and Lee (2013)	Facebook; Moodle 2.0	Encourages students to start their own businesses	to would-be investors. Facebook is commonly used to augment instruction	Students from fourth year elective classes in entrepreneur management	188	Control group experimental study; Questionnaire; Statistics	Knowledge in partner trust and cooperative	Weak
	Ali et al. (2017)	Facebook	Gives students the exposure in developing their skills and interest in business	Social networking sites have been issues; the increasing knowing the powerful of social networking to studies; interrupt engagement	Diploma students in the field of engineering/science	400	A cross-sectional survey by using questionnaires to collect data and SPSS to analyze data	take the initiative; develop a business independently; invest with own capitals; business yields profit	moderate
	Swaramarinda (2018)	ICT	No definition	Teachers should be able to apply ICT; the ease of learning process and technology support teaching and learning	Entrepreneurship teachers	102	Descriptive quantitative survey	Entrepreneurship teachers need creative, innovative and productive learning processes	weak
	Menkhoff and Bengtsson (2012)	Wikis; mobile phones	know and understand discuss the challenges	What is often overlooked by university teachers is the potential of these mobile technologies to provide an interesting and enriching learning experience	Undergraduate and instructors	49	Case study	Students were encouraged to expose themselves to interesting locations	Moderate +
	Wu et al. (2018)	PowToon (web-based ICT)	No definition	ICT is used to traditional teaching methods and competency training	EMBA and MBA	45	quasi-experiment design and qualitative methodology	a business idea	moderate
	Akhmetshin et al. (2019)	Internet, e-mail; website;applications	Develop core business knowledge and skills; core competencies	distance learning has gained popularity; students still need to attend colleges to take accreditation exams	Distance students and FTF students	5	Case study	Knowledge and skills, competencies	Moderate-
	Josien and Sybrowsky (2013)	eBay	New venture creation; ideas; creating new enterprises and jobs; nurturing the economy	EE has received a lot of attention lately; However, conflicting ways on how and what needs to be taught in such classes have emerged	Undergraduate entrepreneurship students	12	A pre-test post-test study	Entrepreneurial skills and aptitude	Weak
	Protopsaltis, et al. (2013)	SGs and platforms	Creative innovation; what factors influenced their success or failure	StartUp_EU	European secondary school students (age 14–18)	47	Pre-post questionnaire survey	Entrepreneurial skills; creating an elevator pitch	weak
SGs	Hauge et al. (2013)	SGs	Starting and managing a business	SGs in higher education (HE) are still quite low; A lack of papers describing deployment; critically showing their educational benefits and providing guidelines and practices	Electronic Engineering B.Sc. and, mainly, M.Sc. students	3 games	a qualitative analysis (case study)	address the field of entrepreneurship (motivation and company management)	moderate
	Bellotti et al. (2012)	SGs	Personal abilities; new products and services; factor for societies	The lack of a common framework for describing/classifying the educational interventions in a SG	Students; instructors; entrepreneurs	41;10;5	Interviews and surveys	entrepreneurship attitudes, knowledge and skills	moderate
	Bellotti et al. (2014)	SGs	Personal abilities; new products and services; a key factor for contemporary societies	EE is still relatively immature and rarely adequately addressed in particular in the technical universities	Higher education engineering students	11	Case study	entrepreneurial mindset	moderate
	Antonaci, et al (2015)	Two EE models	how to run a business; knowledge; skills; attitudes	Lifelong Learning Erasmus Fostering Excellence; Innovation in HE (FEXI)	University students	No sample	Case study, build a model to analyze 9 games in 3 universities	knowledge; skills; attitudes	moderate
	Fox et al (2018)	SGs	A complicated education; innovations and variety in teaching methods	There is a void between current theoretical understanding of the entrepreneurship process	Students	8 (games), 5 (cases)	Systematic literature review; case study	expand knowledge and understanding of educational practice	Strong
	Newbery et al. (2016)	SGs	The process of learning to discover and exploit opportunities	SGs are playing significant role; SGs can provide with an authentic learning experience and increasingly taken up by business school	Undergraduate; business and management students	263	A pre-test/post-test quasi-experiment	Entrepreneurial mindset	Moderate
	Mayer et al (2014)	SGs	Associated with education in other areas	An authentic, experiential didactic that seems particularly appealing to the Net-generation	A master level course in E	28	A quasi-experiment	Enterprising personality, motivation and intentions	Moderate
	Buzady and Almeida (2019)	FLIGBY	How to run a business	This approach allows students to understand entrepreneurial activity; not allow students to understand the consequences	Higher education students	18 (business course);31 (computer science course)	Case study; quantitative survey	Skills in an immersive way and based on real challenges that can be found in business environments	Moderate
	Fellnhofer (2018)	SGs	Change individuals’ attitude toward a career as entrepreneur	There is actually very little experience or proof games as a method for promoting and teaching	EE gamers and gamers	41	Control group experiment and questionnaire survey	Entrepreneurial mindset and start a business	Moderate
	Ruiz-Alba et al. (2019)	SGs	From E aspects, Entrepreneurship is to start a business	Gamification and entrepreneurial intentions still lack empirical investigation	Online courses students	220 respondents	A quantitative research strategy tested before and after gamification experience	Entrepreneurial intentions	Moderate
	Williams (2015)	SimVenture	Mindsets; behaviors; capabilities in young people; start ventures	Evaluating the characteristics and features of games without assessing the benefits to students	Undergraduate management students	32	Action research	Skills, attitudes and behaviors	Weak
	Chandra and Leenders (2012)	Second life	No definition	Scant attention for user innovation and user entrepreneurship that take place within the virtual world	Second life residents	4	Virtual participant observation; interviews	Skills	Moderate
	Mennecke, Mcneill, et al. (2008), Mennecke, Hassall, et al. (2008))	Second Life and e-learning	Expose students to business and e/commerce concepts; start and run businesses	Second Life (SL) boasts more than 15 million accounts and is marked by the presence of a strong educational community	Graduate students	29	Comparative method	Start and run virtual businesses in a manner quite similar to the way people engage in business	Moderate
	Grivokostopoulou et al. (2019)	3D virtual worlds	Boost employment; sustainable development; economic and social development	“Entrepreneurship Action Plan 2020”; the formulation of EE frameworks; challenging domain	Higher education students	86	Pre-post tests using questionnaire	Entrepreneurial mentality, skills and competencies	Moderate
	Wu and Song (2019)	SGs; social media	Differs from that of general subjects. E is fascinating but challenging	Relevant studies have focused on the use of one or two social media platform; three social media platforms have been limited	College students and entrepreneurs	458	Questionnaire survey and interview	Motivation; skills; engagement; knowledge	Moderate
	Vorbach, et al. (2019)	MOOCs	Designing, launching and running a new business	Parallel to the evolution of academic entrepreneurship; rapid acceleration of digital technologies	Higher education students	40	Questionnaire survey of empirical study	Entrepreneurial attitudes, entrepreneurship mindset; knowledge	Weak
	Romero and Usart (2013)	SGs and MOOC	Requires active; engage in activities	Increase the entrepreneurship orientation; MOOCs	Students and adult citizens	76	Case Study	entrepreneurship basics; MetaVals practice knowledge; HotShot Game E competes and skills	moderate
	Resei et al. (2018)	MOOCs	An important area; relevant in times of crisis and economic challenges	Online EE has strongly accelerated in the last two decades by the development of information technologies	Learners	5 platforms	Desk research	Successfully launch a business; international new ventures; developing knowledge and skills	Moderate
MOOCs	Cirulli et al. (2016)	MOOCs	Incorporating businesses; alternative business models	MOOCs are changing the way in which people can access digital knowledge; creating new opportunities	Higher education students	10	Interviews	Behavior; benefits and opportunities for both individuals and organizations	Moderate
	Al-Atabi and Deboer (2014)	MOOCs	Innovation; technological progress, economic growth	Entrepreneurship as a skill and process is increasingly being taught as a part of various university educational projects	Engineering students	80	Questionnaire survey	collaborative learning; opportunity recognition and resource acquisition	Strong
	Solórzano-García and Navío-Marco (2019)	MOOC communities	Tools, skills and resources to develop their projects	Provide them with a learning environment that gives them the opportunity to be social entrepreneurs	Learners	3250	Pioneer social entrepreneurship massive open online learning communities	Start a business	Moderate +
	Kakouris (2016)	TeleCC.org	Entrepreneurial learning in educational terms	Entrepreneurial learning leaks into informal, non-academic settings either face to face or online	Postgraduates and online learners	18(F2F), 22(online)	Questionnaire	Knowledge; skills, not focus on start a business	Weak
	Frederick (2007)	Moodle	Focusing on realization of opportunity on the best way to operate hierarchies	The technology-savvy generation under the motto “Teaching is best done online and learning is best done in the classroom”	Generation Y	No sample	A grounded theory approach	Theory, process and practice, commercialize their ideas	Moderate
	Wu et al. (2019)	Mobile-based CRS (ZUVIO)	A key role in pursuing EI to provide a highly qualified entrepreneurial workforce	Traditional CRS may exhibit difficulties; the use of mobile devices and wireless technologies in education was increasingly	Graduate students	22	Qualitatively; reflection learning report; questionnaire survey	Business knowledge; the art of entrepreneurial experience	Strong
	Chang (2017)	World Cafe ´ forum;BOSS	Practical knowledge related to the establishment of new business ventures	Many teachers involved in entrepreneurial training are now implementing the ‘‘World Cafe ´’’ strategy to augment traditional classroom discussions	Participants were hoping to start their own business within 3 years	120	Questionnaire survey	Skills (write business plans)	Moderate

*EE* Entrepreneurship education

A variety of digital technologies in the online and blended environment have been adopted into EE such as cloud computing (Holinska et al., 2019), learning analytics (Toledo et al., 2020), there digital (3D) virtual reality, SGs (Lameras et al., 2015), social media, digital platforms, big data (Secundo et al., 2020; Sousa, 2019) and so on. The emphasis of these works has been on the implementation, and lack of appropriate or relevant research on the pedagogical and usability aspects. The results of our systematic review showed that the first study on online and blended EE was conducted in 2006 (Arbaugh et al., 2010). Commencing from 2010, entrepreneurial courses have been made available on MOOCs platforms in cooperation with different universities and there have been a number of SGs developed by the games industry to enhance EE. Before the pandemic, learners may have still preferred the F2F format compared with the online and blended version, despite the increased popularity in the latter. However, during the pandemic where the availability of courses were only limited to online, there has been an increased interest in the online and blended EE courses.

### Social media in EE

Social media is a method of Web 2.0 that places emphasizes on the exchange of views with other learners (Jones & Iredale, 2009). A majority of the new generation born in the digital era embrace social networking sites (SNS) and often fill their daily lives with communicating with others via social software, which is perceived as a welcome way of building distributed human interaction. Additionally, college students adopt social media to informal and formal learning (Dabbagh & Kitsantas, 2012), increasing student engagement (Blaschke, 2014) and satisfaction (Barczyk & Duncan, 2012). Most importantly for entrepreneurship learners, one aim of taking an EE course is to build a social network and human relations (Man et al., 2002; Mitchelmore & Rowley, 2013), where participants communicate with each other (e.g., Facebook, Twitter and WhatsApp) and show their life (e.g., Snap Chat, YouTube and Instagram) and work experiences (e.g., LinkedIn, Facebook and ResearchGate) in social media sites and applications. Kaplan and Haenlein (2010) classified social media into two branches: social presence and media richness and self-presentation and self-disclosure. Self-presentation (e.g., personal profiles) is the impressions that other users form on the user. It is one basic function of social media and has a connection with conversation and relationship (Kietzmann et al., 2011). Since this study specially analyzed SGs which overlaps with the taxonomy of Kaplan and Haenlein (2010), we excluded them in this section and classified papers (n = 9) using social media technologies into four sub-areas: the extent of media richness (namely, low and high) and interaction level (namely, weak and strong), which is shown in Table 2.

**Table 2:** Classification of social media in entrepreneurship education by media richness and interaction

		Media richness	
		Low	High
Interaction	weak	Collaborative projects (e.g., Wikis, podcast and blog)	Content communities (e.g., YouTube and eBay)
	strong	Forums (e.g., Moodle forums)	Social networking sites and applications (e.g., Facebook, Skype, WhatsApp and Twitter)

When designing a course, an animated business plan presentation tends to give better and more attractive results than a presentation without animated videos (Wu et al., 2018). Video reflection is a supplement teaching tool for written reflection (Wraae et al., 2020). Based on the benefits of different social media applications, educators introduced text-based, audio-based and/or video-based versions into students’ learning processes. It is noted that schools were ready to utilize information communication technologies, whereas teachers themselves still lack the readiness (Swaramarinda, 2018). In EE, social networking sites are supplementary technologies for interaction and communication, More specifically, bachelor students using a wiki (namely, *Wetpaint*) to create and edit e-commerce websites together through brainstorm (Barczyk & Duncan, 2012). Learners can communicate with other learners, entrepreneurs and entrepreneurship consultants in the Facebook and the Facebook community when attending a business planning course (Chang & Lee, 2013). YouTube is used to post presentation videos in business education (Alon & Herath, 2014). Online students record and upload an elevator pitch video to YouTube in entrepreneurial management open by Royal Roads University. The website of eBay also works as an experimental learning tool. Students upload their information of goods and consumers bid on the website (Josien & Sybrowsky, 2013). In *Internationalization of Entrepreneurial Marketing Education* courses, students located in three different countries often met and conducted many of their teamwork activities in a virtual environment through the utilization of Skype, WhatsApp and other social media tools (Reid et al., 2018). *The Chinese Entrepreneurial and Asian Business Networks* course is taught in a Singapore university where learners utilized *Mediawiki* and SNS to send messages to each other on their mobile devices (Menkhoff & Bengtsson, 2012).

SNS promotes sharing learning materials and resources, like sharing photos on the Flickr site (Menkhoff & Bengtsson, 2012). Also, personalized content service is provided by utilizing social media that facilitates self-regulated learning (McLoughlin & Lee, 2010). Besides, social media usually combine with digital educational platforms (Chang & Lee, 2013; Waghid, 2017), augmented reality (Gupta & Bharadwaj, 2013), or work as a technology-enhanced learning environment (Manca & Ranieri, 2013a, 2013b) to facilitate EE. Since social media are only a means of digital technologies, digital platforms, big data, intelligent applications, digital storytelling and other digital methodologies are applied to EE together (Secundo et al., 2020; Sousa, 2019). To conclude, as a vital communication method, social media use in daily life and workplaces and education scenes. Therefore, users are familiar with social networking tools that are easy to accept and adopt. Social media provides text, audio and video message and information for online and blended entrepreneurial learners. Instructors and learners consequently have the initiative to choose an appropriate medium. Hence, the interaction and connections between learner-learner and instructor-learner increase the benefits of active learning and social capital (Gupta & Bharadwaj, 2013).

### Serious games (SGs) in EE

The idea of SGs lies in the utilization of games and gaming technologies edutainment: not only entertainment but also education and training (Eck, 2006). In other words, SGs bring to learners additional enjoyment (learning by playing or gaming) and simulate different scenarios to enact real-life situations (Susi et al., 2007). Learning entrepreneurial skills via real-life business scenario simulation can avoid and limit real-life risks and damages, reduce the cost when acquiring entrepreneurial skills and competencies. Concerning learning objectives, as opposed to entrepreneurial knowledge (e.g., finance and marketing), SGs develop with more attention toward facilitating entrepreneurial mindset and competencies, especially in innovation, opportunities spotting and risk management (Almeida, 2017). In the second set of articles, 20 of these focused on the effectiveness of SGs in EE. Based on the demand for innovation and experiential entrepreneurial competencies (Constanţa-Nicoleta et al., 2015), game designers simulate a real business environment for learners to run a virtual business (Mennecke et al., 2008, 2008) and avoid risks in the real world so that the cost and uncertainty of being entrepreneurs decrease. Except for general effectiveness (Tasnim, 2013), scholars focused on the entrepreneurial mindset, entrepreneurial intention and motivation (Buzady & Almeida, 2019; Ruiz-Alba et al., 2019), entrepreneurial behavior (Fellnhofer, 2015) and entrepreneurial competencies (Williams, 2015). Mayer et al. (2014) narrowed the research subjects into engineering students, which showed gaming experience could significantly influence EE. Furthermore, Bellotti et al. (2014) analyzed the entrepreneurial mindset of engineering students. In general, the relationship between SGs and entrepreneurship intention and skills is positive (Almeida, 2017; Bellotti et al., 2014; Buzady & Almeida, 2019; Fellnhofer, 2015; Ruiz-Alba et al., 2019), even long-term positive effectiveness (Kriz & Auchter, 2016). However, the study of Newbery et al. (2016) found a significant negative impact for the authentic learning method, perhaps because students understood the complexity of starting a business (Protopsaltis et al., 2013). Fox et al. (2018) conducted a systematic review on SGs of EE and evaluated games from fidelity, verification and validation in entrepreneurial learning. They argued that SGs had practical value for authentic learning and should be introduced to learners before learners start a business in real-life. However, the real business environment is more ambiguous and lacks nonplayable characters which appear in in-game worlds (Fox et al., 2018), more complex and uncertain than the virtual business world. Entrepreneurial games still lack complexity, uncertainty and interactivity at present to avoid life lessons needed to be experienced by entrepreneurs.

Specific SGs have been applied to EE. Cluster analysis was applied to game-play experience from 7 aspects to compare *TeamUp*, *Slogan* and *SimVenture.* Compared with *TeamUp* and *Slogan*, *SimVenture* is rich and complex for learners (Mayer et al., 2014). These entrepreneurial games are still found: *GoVenture Card Game*, *the Entrepreneur Card Game*, *GoVenture: Entrepreneur* and *Monopoly*. Non-digital games are applied in the F2F class, i.e., *Monopoly* and *Slogan*. In an online and blended learning environment, digital SGs, e.g., *FLIGBY* and *SimVenture*, are growing in popularity at all school levels. Through reviewing the literature, *Hot Shot Business*, *SimVenture*, *ENTRExplorer* and *FLIGBY* attracted much more attention from scholars and educators in the 2010s. *Hot Shot Business* is a computer-based entrepreneurship game that was developed by Disney and had been dropped from the website. *SimVenture* (www.simventure.com) has two simulation games: *SimVenture Classic* and *SimVenture Evolution*. As a part of undergraduate and postgraduate modules, *SimVenture Evolution* is applied in 10 UK universities and colleges, following the principle of ‘learn by doing’ (Williams, 2015). *ENTRExplorer* (https://www.entrexplorer.com/projecto.php) is for immersive entrepreneurs funded by European Commission, simulating a business through 3D and multiplayer. *FLIGBY* (http://www.Fligby.com) is a web game, especially for leadership learning. Buzady and Almeida (2019) analyzed the function of *FLIGBY* from 29 indicators, which shows both technical skills and soft skills increased after playing. To adopt an appropriate game, Antonaci et al. (2015) introduced three strategic axes as well as target skills and pedagogical/usability features for instructors and scholars. Educators adopted several SGs for different teaching objectives and context at one online EE, such as business plan, a pilot project of the entrepreneurial idea, market and product analysis as well as evaluation of entrepreneurial skills (Sousa, 2019) course. Concerning the amount of applied SGs, Romero and Usart (2013) utilized two games (*Meta Val*s and *Hot Shot Business*) to help learners learn entrepreneurship. Bellotti et al. (2014) analyzed three games (pre-, mid- and post-game) in one-course time. Hence, according to the objectives of online EE and phrase, instructors provide plenty of games in class or at home for learners (Antonaci et al., 2015).

SGs are found in MOOCs platforms to increase experiential learning activities as well (Romero & Usart, 2013). In the web 2.0 learning content management system, each game connects with a specific entrepreneurial task and mini-games are independent of the system (Protopsaltis et al., 2013). Today, SGs simulate a virtual world characterized by avatars and a 3D environment. In a virtual social world, almost every facet imitates real life. For example, gamers start a business, communicate with other avatars and earn virtual currency in *Second Life* where they acquire a notion of the entrepreneurial process in an e-commerce course (Mennecke, Mcneill, et al., 2008, 2008), whereas establishing too many rules will restrict avatars and lead to low self-presentation in a virtual world (Kaplan & Haenlein, 2010). In other words, even virtual reality technology or other technologies only simulate the real business, it still a simulated process of being entrepreneurs, which is simplified and idealized. In addition, aims and the phrase of EE courses are the essential consideration for participants to find out the most suitable ones among the present and constantly designed entrepreneurial games. However, we still lack standardized methods and metrics to choose appropriate entrepreneurial SGs. Furthermore, the future trend of SGs will continue to combine with other cutting-edge technologies to simulate starting a business and entertain the process of learning.

### Digital platforms in EE

As a digital educational platform, a course management system or student management system restores and manages data of learning materials, students’ performance and interaction data. When starting a discussion, the system automatically distributes questions and team leaders in online World Cafe ´ (Chang, 2017). The platform facilitates interaction between student–teacher/peer and learning engagement (Wu et al., 2017). Most online learners have sufficient time to finish EE courses and express ideas freely (Kakouris, 2016). EE courses on MOOCs platforms are open, free and many pay a small amount of tuition to get a certificate (e.g., Most courses cost less than 100 euro to get a certificate). Whilst educators and learners can share high-quality learning resources around the world, which is one of the most obvious advantages of digital platforms. Many stellar universities and companies cooperate with MOOCs platforms to upload entrepreneurial courses and resource in the version of the text, audio and video. Almost all of the MOOCs platforms are on-demand video lectures, playing on a mobile phone, tablet and other devices, providing flexible deadlines and self-paced learning (e.g., Udemy). While UNX provided MOOCs (courses are linked with Udemy platform now) for entrepreneurship and community for entrepreneurs or future entrepreneurs mainly in Spain, Portugal and Latin American (Piñuel, 2014), many MOOCs (e.g., Coursera and EdX) platforms set up “entrepreneur or entrepreneurship” sub-model for worldwide learners. Based on the summarization of Baturay (2015) and information on related platforms, 7 common MOOCs management platforms provide EE courses and resource through the US and Europe. The detailed information is shown in Table 3 which authors used “entrepreneurship” to collect EE courses until Feb. 25, 2020.

**Table 3:** MOOCs platforms providing entrepreneurship courses

Platform	Learning products	Level	Filter criteria	Language	
Coursera	Courses (192), Degrees (3), specialization (28), Mastertrack™ Certificate (1)	Beginner (111), Mixed (78), Intermediate (31)	5	31	
EdX	Courses (59), programs (51), certificate (31), MicroMasters Programs (15), Professional Education (31), Verified (59), XSeries (5)	Introductory (43), Intermediate (19), Advanced (2)	6	English Spanish	It shows availability of these products. Learners easily find the appropriate
Udemy	No given	All levels (1834), Beginner (1204), Intermediate (207), Expert (32)	9	9	Ratings, price need paid or not
Udacity	Free courses (2)	Beginner, Intermediate (2), Advanced	3	English	
Iversity	Courses (2), Programs (2)	No given	3	English German	
MiriadaX	Courses (3)	No given	0	English French	
Futurelearn	Courses (39), Degrees (8), Career advice (1), Partners (1), Topics (1)	No given	1	English	

The first three platforms, namely, Coursera, EdX and Udemy have the majority of online EE courses. To quick select suitable courses for learners, based on learners’ entrepreneurial learning background, platforms set filter criteria for learners to narrow scope of courses and easily have a suitable start. Since MOOCs are open to learners worldwide, platforms prefer providing English and giving languages options as many as possible. Udemy has the largest amount of entrepreneurship learning products and filter criteria, compared with other 6 platforms. According the components of distance EE courses, at present, it mainly contains on-demand videos, reading materials, exercises, discussion forums, test as well as learning dashboard designed. The content of video focuses on entrepreneurial knowledge framework and skills as well as interview video of successful entrepreneurs. To make MOOCs sustainable development, micro-credentials (acquisition of specific skills) and university credits (Resei et al., 2018) are introduced into MOOCs platforms. Karma, namely digital reputation, is a factor of retention and completion rate related with learners, rewards and interaction (Navío-Marco & Solórzano-García, 2019). MOOCs combine with interaction to reduce high-rate dropout, e.g., badges, forums, and on-campus students invite online learners to join their teams. Skeptics argue that MOOCs platforms are lack of F2F communication, frequent feedback (Welsh & Dragusin, 2013) and self-discipline to complete courses (Romero & Usart, 2013; Vorbach et al., 2019).

Although a large number of courses resource have high enrolment are presented, 5% course completion rate is typical (Jordan, 2014). A high dropout rate may be unsatisfied with previous online learning experience lack of support services (Ifenthaler & Yau, 2020). While MOOCs are still the mainstream method to construct online and blended entrepreneurial courses (esp. cheap, easy to access, sophisticated framework and established courses). Hence, we attempt to analyze several common educational technologies introduced into EE, combine with online and blended education to meet the needs of learners for high-quality online entrepreneurship courses and boost course completion rate on digital platforms. In conclusion, a digital system is a choice for spreading EE. Universities and corporations uploaded EE courses on platforms for distributed learners to get entrepreneurship—related micro-credentials and degrees. Therefore, instructors and learners easily access worldwide high-quality EE resources without limitation of time and space. While lack of F2F interaction and communication weakens the effectiveness of EE. Therefore, except forums, lecture videos and text materials, platforms are supplied by social networking software (Frederick, 2007; Solórzano-García & Navío-Marco, 2019), SGs (Romero & Usart, 2013) and other technologies.

## Discussion

As online and blended entrepreneurial educators, learners and scholars involved, it is necessary to master the advantages and challenges of social media, SGs, digital platforms and their combination. Nevertheless, reviews of educational technologies in online and blended EE are still lacking, especially a comparison between them, probably because the F2F method of EE has been dominating the trend. Today, for the pandemic, EE has to transfer into an online and blended environment. Instructors and stakeholders consider educational technologies to facilitate the effectiveness of learning and teaching. Hence, it is time for a systematic review and compares those three technologies mentioned above.

### Social media

Social media is a complimentary resource in professional work (Gruzd and Goertzen, 2013), management education as well as EE (Rueda et al., 2017), since potential entrepreneurship connections and social networks are a success factor for future entrepreneurs. In Italian CLabs, social media is the most leveraged one, compared with big data, digital platforms and other digital technologies (Secundo et al., 2020). learners share learning materials and resources, do teamwork collaborating with others and discuss questions (Ajjan & Hartshorne, 2008; Mazman & Usluel, 2010) using various information and communication technologies (ICT) (e.g., text, pictures, audio, video, or a combination). Instructors’ effective attendance in social media make better teaching performance (Gruzd et al., 2018), accords with “teacher presence” in an online learning environment (Garrison and Anderson, 2003). However, tutors and chatbots will be a more common alternative for timely feedback because of the overload communication tasks (Fryer et al., 2019). The main function of social media is supporting learning and self-managed learning through boosting learner-to-learner and learner-to-teacher interaction, which produces many short-term teams (namely, the aim of the team is mainly for EE and teammates are not active after completing the course) and leads to collaborative learning. Liu et al. (2010) explored the Perceived Variables to Technology Acceptance Model (TAM) to research students’ intention to use an online learning community. There are five factors (trust, mutual influence, conflict, leadership and cohesion) that impact student knowledge sharing within virtual teams through the synchronous and asynchronous communicational environment (He & Huang, 2017). Developing trust, making learning contract and making sure membership role differences (Allan & Lawless, 2003) are necessary for online and blended EE.

Social media with low media richness and weak interaction usually is applied to learn entrepreneurial knowledge. Podcast records short course-related instruction audio for learners’ mobile learning. By assigning tasks to every teammate or a team, Wikis content is created by students with a guideline. SNS with lower media richness frequently cooperates with other social media and users communicate asynchronously. For example, learners asynchronously communicate on forums and exchange materials so that their classmates and subsequent learners can learn from existed communication information. The recordings of communication can be leveraged for learning analysis. Learners’ comments on the blog were collected to conduct inductive reasoning and analyze the learning effect. To make the congruence between comments and students’ reality, student interviews were added (Waghid & Oliver, 2017). Social networks with strong interaction usually share profiles, information and ideas, which probably build a personal relationship and human network for starting a business. As an example of strong interaction and high media richness, a Facebook community can improve the learning effects of writing a business plan through increasing understanding of partner trust and cooperative learning (Chang & Lee, 2013). Compared with other social tools (e.g., WhatsApp, Line and Twitter), Facebook increased learners’ participation even in cross-cultural communication. For example, a Facebook page encourages students to post and follow learning tips so that it facilitates external interaction (Divall & Kirwin, 2012). Besides, Facebook is an important teaching instrument but not a unique one (Manca & Ranieri, 2013a). Compared with low rich social media, entrepreneurial learners perceived that Facebook is more popular and effective (Swaramarinda, 2018). However, as reported, Facebook is the fourth most popular social platform for American’ youth, compared with YouTube, Instagram and Snapchat (Anderson and Jiang, 2018). In other words, video social media are increasing. Educators should be open to new social media to consider the application possibility of entrepreneurial pedagogy.

Compared with F2F, educators are difficult to get real-time learning feedback in online and blended learning environments, especially in MOOCs platforms. With the application of SNS, learners easily communicate with each other and educator-learner have more connections. Learning devices easily record and collect learning data of communication, especially plenty of attendees in online learning, which leads to learning analysis that is a technological tendency of the 2020 Horizon report in higher education.

### Serious games

SGs leveraged in EE have the practical experience and theoretical basis. The project of ‘Stimulating Entrepreneurship through Serious Games’ (eSGs, 2011–2013) was executed in four universities of three countries (Bellotti et al., 2012). As a member of this project, Mayer et al. (2014) analyzed the function of SGs and factors that determine its contributions at Delft University of Technology through qualitative and quantitative methods. Depending on collecting data, which is produced by gamers from devices, SGs are sensitive to analyze data of results and update the functionality to meet the players’ requirements and facilitate active learning in time.

SGs make EE courses more interesting and attractive than traditional lectures. However, this doesn’t mean the motivation of starting a business will increase after playing a game, even the motivation of females decreased (Kriz & Auchter, 2016). What cannot be denied is simulating the process of entrepreneurial activities is to identify business opportunities and start-up and marketing strategies (Constanţa-Nicoleta et al., 2015). Simulative games make this process attractive. e.g., gamers attend entrepreneurship activities from *Second Life* which is a simulative business game and they can even use virtual money in the virtual life. Educators encourage and purchase an island on virtual *Second Life* space for learners to play this game (Mennecke et al., 2008, 2008). While many SGs are stiff and rigid to play with. For example, gamers must follow step by step or skip several steps and they are hard to follow their innovative ideas (e.g., *Hot Shot Business*). Therefore, compared with other video games, simple SGs are not interesting enough and cannot meet their needs. Today, developers have produced more authentic roles in *FLIGBY* and *SimVenture* to make games interested and real, such as 3D, simulated market and multi-players. Consequently, students can acquire learn entrepreneurial skills and behavior more authentically. Educators choose appropriate SGs as teaching tools depending on entrepreneurial teaching objectives and characteristics of games (Antonaci et al., 2015). Also, the application of SGs needs is wider to structure and amend criteria of assessment. Besides, participants and stakeholders pay close attention to technology development directions and trends to apply by SGs. With the expansion of AI, it has been adopted by SGs (e.g., the virtual game world) to facilitate an immersive and virtual learning environment. SGs combine with new technologies, MOOC platforms and social media, which is the near-future scenario. In the virtual game world, players apply social media to their virtual world communication. As an indispensable component in the virtual game world, social media might instead of F2F communication in a virtual learning environment.

### Digital platforms

Learners easily access high-quality entrepreneurial resources, because the collaboration between digital platforms and HEIs makes online EE courses professional and low cost. In contrast with the huge number of learners, the completion rate of entrepreneurial courses on digital platforms is relatively low. For one reason, the competence of self-discipline learning and the strategies of setting own learning pace are necessary for distributed learners. The other main reason is entrepreneurship competence and mindset are achieved through practical activities and interaction amongst learners, which digital platforms are still lacking. Consequently, most EE courses provide online discussion forums to supply online interaction and connection. Instructors usually give topics related to the course to discuss and learners post their puzzles, which improves cooperative and collaboration competence and reduces the dropout rate of courses. All participants with accounts and passwords easily log in to the platform and look for existing entrepreneurship learning resources. Instructors set the “introduce yourself” or “know your classmates” section to know basic information about learners. What’s more, instructors appear in the discussion section and their attendance is highlighted (e.g., Coursera). Compared with forums, SNS supports timely contact and feedback. Thereby, digital platforms introduced social media as well, especially in cMOOCs. cMOOCs focus on connection, emphasize social networking and are based on the philosophy of connectivism (Rodriguez, 2013). Social media being another main learning method in cMOOCs comment and enhance interactions and collaboration among global virtual classmates (Kaplan & Haenlein, 2016). For example, *Identifying Entrepreneurial Opportunities* by the University of Maryland on EdX provides an extra social media link for learners to know each other. SGs are applied to MOOCs, which make for shortcomings of it, such as engagement (Freire et al., 2014), completion rates and motivation (Borras-Gene et al., 2016) and so on. SGs depend on or are independent of MOOCs platforms. SGs which have a close connection with MOOCs platforms need to give feedback beyond “global outcomes” (Freire et al., 2014) and trace multi-level assessment and individual actions to collect more detailed learning data. Cooperating with curriculum designers, SGs which are independent of MOOCs platforms build their platforms. Meanwhile, MOOCs platforms provide an entrance or link for players to log in to games.

In summary, with the rapid development of MOOCs in the 2010s, many digital platforms provide entrepreneurship videos, exercises and learning materials, combined with forums and workshops, which boost learners international collaboration (Welsh & Dragusin, 2013) and affect behaviors and skills related with entrepreneurship (Calvo et al., 2019). MOOCs accelerate the accessibility of EE because of flexibility in time and space (Vorbach et al., 2019). Meanwhile, MOOCs platforms provide EE credentials and degrees based on learners’ performance to facilitate completion rate (Resei et al., 2018). While the low completion rate of MOOCs needs designers of course provide more support services. Hence, MOOCs platforms flexibly harness social media, SGs and other technologies.

### Comparison between the three technologies

The applications of social media, SGs and digital platforms are comparatively broadly in EE. Evaluating and scoring them depends on the usefulness shown in Table 4. These quality criteria were based on Nielsen (1993), who classified usefulness into usability and utility. Analyzing technical usability (sub-concept of usability) (Hindle, 2002) is easy to master, efficient, easy to remember, few serious errors and user satisfaction. The Utility is whether one can address the needs of the user (Nielsen, 1993; Nokelainen, 2006). Pedagogical usability is a sub-topic of utility. The highest got score 3, the middle got score 2 and the lowest got score 1. Social media were classified into *Wiki* and *Facebook*. *FLIGBY* and *SimVenture* are illustrations of SGs. Coursera is a research example of digital platforms. Based on every built criterion, the aforementioned five items scored higher than 30 points. From entrepreneurship learning aspects, compared with social media and MOOCs, SGs simulate authentic business scenarios in which learners learn by doing to facilitate entrepreneurial motivation, mindset, competence and participation rate. While SGs got a lower score for lack of systematic design in entrepreneurial knowledge. Whilst Coursera has a good performance in acquiring knowledge. Except for the flexibility of methods, Coursera got the highest score in the teaching area, partly because learning on digital platforms has large similarity with traditional education which educators have a profound experience of didactics. Social media has the best performance in the interaction and cooperation part. Facebook as a social communication tool easily build relationship amongst distributed users. From technical usability aspects, participants are easy to master social media and they need to learn rules to play SGs. Besides, how to use social media is the easiest to remember for users, especially the young generation (namely, Y-generation and Z-generation). However, SGs are the most efficient of the three and their users’ satisfaction is the highest, which consistent with the essence of games. Compared with the high error risk of SGs, MOOCs have few errors, since MOOCs need lower-level technology support than SGs.

**Table 4:** Comparison of three technologies applied in entrepreneurship education

			Social media		Serious games		Moocs
			Wiki	Facebook	FLGBY	SimVenture	Coursera
Pedagogical usability	Usability of E learning	E motivation	2	2	3	3	1
		E knowledge	2	2	1	1	3
		E competence	1	2	3	3	1
		E mindset	1	2	3	3	1
		Participation	1	2	3	3	1
	Usability of E teaching	Efficient of E guideline	2	2	2	2	3
		Flexibility of E methods	1	1	2	2	1
		Quality of E activities	2	2	2	2	3
		Achievement of objectives	2	2	1	1	3
	Usability of interaction	Tutor–student interaction	2	2	2	2	1
		Students interaction	2	3	2	2	2
		Student-entrepreneur interaction	2	3	1	1	2
Technical usability	Easy to master		2	2	3	3	2
	Efficient		2	2	1	1	3
	Easy to remember		2	2	3	3	1
	Few errors		2	2	1	1	3
	User satisfaction		2	2	3	3	1

Therefore, social media, SGs and digital platforms have been the most popular technologies applied to online and blended EE until now. All three depend on technology devices to store detailed learning and teaching data that are learning analytics objects. Meanwhile, these technologies are incentive to cutting-edge technologies to update themselves. Social media provide tools to share information, do teamwork and ask and answer questions without being restricted by time and space in EE. SGs make EE more interesting and attractive as well as games simulate real business and reduce costs. MOOCs provide worldwide, free or low-cost learning possibilities. Courses combine social media with SGs to facilitate collaboration and effectiveness. Gamification factors also are added to social media (Wu & Song, 2019).

This study gathered 121 related to English literature and reviewed 38 published papers. One limitation of this research is still the lack of data. Although the combination between EE and educational technologies is a potential area, the application still is a fresh and ever-changing domain. With the rapid speed of technology development, technicians are adopting another technology possibility to develop application. This research mainly concerns about three relatively mature technologies without those new technologies. The other limitation is the classification of the learning environment. As lacking literature to delimit different terms of learning environment, this research summarizes “online and blended” learning instead of other learning environments.

## Conclusion

Entrepreneurial competence is critical for individual and economic entities. Furthermore, nowadays both in developed countries and developing countries, the knowledge society calls EE in all levels of education, especially in HEIs. Educational technologies accepted by management education have been reviewed in the last two decades worldwide, while educational technologies applied in EE lack systematic review, especially in online and blended entrepreneurial learning as well as teaching. The present study aims to systematically review three popular technologies used in EE and evaluate the effectiveness in the online and blended learning environment through a comparative method. Compared with the F2F or traditional learning environment, the online and blended EE breaks time and distance limitations. Online learning is a broad definition, which contains e-learning, distance learning and mobile learning. Blended learning is a tendency for higher education institutions and learning corporations. Meanwhile, technological support is provided to promote entrepreneurial learning as well as instruction so that many popular educational technologies came into our view. When collecting literatures, social media, SGs and digital platforms are the most popular adopted educational technologies in EE. For the application of three educational technologies is broad in instructors and learners, published literature focuses on those three technologies more than other technologies.

Regarding social media, which prompts interaction between learner and learner (as well as the learner to instructor), it brings the possibility of online learning, especially ubiquitous learning. Compared with educational technologies described in the Horizon Report 2020, social software is mainly used before and after online EE courses for preview and review, since learning platforms and F2F are still the main methods for instruction and active learning in EE. Students' learning data is stored on a computer or other smart devices, which makes it easier to be collected and analyzed than before. Instead of application alone, social media is usually accompanied by other technologies. As technologies develop, social media will be more intelligent and multi-functional for learning analysis soon. SGs make EE more interesting and attractive than courses without SGs. While games simulate real business and base on action orientation, participants learn entrepreneurial motivation, skills and knowledge from experiential scenarios. Learning objectives, phrases of EE courses and learning status are basic considerations for choosing SGs. Meanwhile, scholars should construct and standardize criteria for choosing entrepreneurial games in EE. Participants choose EE courses on MOOCs platforms which have different traits and advantages. Skeptics argued that MOOCs lack F2F interaction, frequent feedback and sufficient support services and self-discipline to complete entrepreneurship courses, while digital platforms facilitate the accessibility of EE because of flexibility in time and distance. And providing entrepreneurial credentials and degrees based on learners’ completion and performance. In light of marking these three educational technologies in online and blended EE, every technology has its own characteristics and appropriate relevant educational scenes.

In general, this study has identified digital platforms that provide worldwide, free or pay little and non-F2F learning possibilities, combined with social media to enhance interaction and SGs to increase engagement, completion rate and motivation. With the appearance of cutting-edge technologies, educational technologies in EE need to update technologies and consolidate theoretical underpinning (namely both technologies and pedagogy). One main objective of educational technologies in EE is facilitating individual and collaborative competencies. This study appears to be the first study to compare social media, SGs and digital platforms used in EE to evaluate the effectiveness and challenges they need to face. For making sure EE and business education more authentic, attractive, convenient, effective and efficient, a further study will focus on the concrete effects of the three technologies with AI to facilitate entrepreneurial collaborative competencies in an online and blended learning environment.
